# hsa-miR29b, a critical downstream target of non-canonical Wnt signaling, plays an anti-proliferative role in non-small cell lung cancer cells via targeting MDM2 expression

**DOI:** 10.1242/bio.20134507

**Published:** 2013-05-22

**Authors:** Sreedevi Avasarala, Michelle Van Scoyk, Jianbin Wang, Marybeth Sechler, Katherine Vandervest, Christine Brzezinski, Colin Weekes, Michael G. Edwards, John Arcaroli, Richard E. Davis, Rama Kamesh Bikkavilli, Robert A. Winn

**Affiliations:** 1 Division of Pulmonary and Critical Care Sciences, School of Medicine, University of Colorado, Anschutz Medical Campus, Aurora, CO 80045, USA; 2 Department of Biochemistry and Molecular Genetics, School of Medicine, University of Colorado, Aurora, CO 80045, USA; 3 Division of Medical Oncology, School of Medicine, University of Colorado, Anschutz Medical Campus, Aurora, CO 80045, USA; 4 Veterans Affairs Medical Center, Denver, CO 80220, USA

**Keywords:** Frizzled 9, MDM2, Non-small cell lung cancer, p53, Wnt7a, hsa-miR29b

## Abstract

In non-small cell lung cancer cell lines, activation of β-catenin independent signaling, via Wnt7a/Frizzled9 signaling, leads to reversal of cellular transformation, reduced anchorage-independent growth and induction of epithelial differentiation. miRNA expression profiling on a human lung adenocarcinoma cell line (A549) identified hsa-miR29b as an important downstream target of Wnt7a/Frizzled9 signaling. We show herein that hsa-miR29b expression is lost in non-small cell lung cancer (NSCLC) cell lines and stimulation of β-catenin independent signaling, via Wnt7a expression, in NSCLC cell lines results in increased expression of hsa-miR29b. Surprisingly, we also identify specific regulation of hsa-miR29b by Wnt7a but not by Wnt3, a ligand for β-catenin-dependent signaling. Interestingly, knockdown of hsa-miR29b was enough to abrogate the tumor suppressive effects of Wnt7a/Frizzled9 signaling in NSCLC cells, suggesting that hsa-miR29b is an important mediator of β-catenin independent signaling. Finally, we show for the first time that hsa-miR29b plays an important role as a tumor suppressor in lung cancer by targeting murine double mutant 2 (MDM2), revealing novel nodes for Wnt7a/Frizzled9-mediated regulation of NSCLC cell proliferation.

## Introduction

Lung cancer represents the leading cause for cancer-related deaths in the world (Jemal et al., 2011; Siegel et al., 2013). Lung cancers, especially, non-small cell lung cancers (NSCLC) display frequent loss of not only tumor suppressor genes like p53 (∼50%), Rb (retinoblastoma protein, 15–30%) and p16^INK4^ (30–70%) but also Wnts, particularly Wnt7a (Sekido et al., 1998). Wnts are secreted glycoproteins that bind seven-transmembrane containing receptors Frizzleds, and stimulate diverse array of morphogenic and developmental-specific programs (Peifer and Polakis, 2000). Wnt binding to Frizzleds, mediated by G-proteins and Dishevelled, leads to post-transcriptional and post-translational mechanism/s-mediated stabilization of β-catenin (Bikkavilli and Malbon, 2010; Bikkavilli and Malbon, 2012; Malbon, 2005). Stabilization of β-catenin allows its nuclear translocation, where it functions as a transcriptional co-activator along with the T-cell factor (TCF) and lymphoid enhancer factors (LEF) (Behrens et al., 1996; Molenaar et al., 1996). Deregulated Wnt/β-catenin signaling leads to many cancers (Karim et al., 2004; Mazieres et al., 2005). In strong contrast, Wnt7a/Frizzled9 signaling was shown to play a protective role in lung cancer (Winn et al., 2005). Interestingly, Wnt7a expression is lost in majority of NSCLC cells (Winn et al., 2005), and restoration of Wnt7a expression in the same cells leads to reduced cell proliferation, reduced anchorage-independent growth, increased cell differentiation and reversal of transformed phenotype (Winn et al., 2005). Previously, we have shown that Wnt7a binding to Frizzled9 (Fzd9) receptor activates a potent tumor suppressor, the peroxisome proliferator activated receptor γ (PPARγ) (Winn et al., 2006). However, the nature of the downstream targets of Wnt7a/Fzd9-stimulated PPARγ and the mechanism/s of Wnt7a regulation of NSCLC cell growth remains largely unknown.

Among the non-coding RNAs, microRNAs (miRNAs) represent a major subset, which regulate target mRNAs either by site specific cleavage or by translation repression (Garzon et al., 2006). Although an estimate, miRNAs constitute nearly 1% of all the predicted genes in nematodes, flies or mammals (Baskerville and Bartel, 2005; Lai et al., 2003; Lim et al., 2003a; Lim et al., 2003b). miRNAs not only play critical roles during development but also during cell proliferation, differentiation, and apoptosis (Calin et al., 2006). The role of miRNAs during oncogenesis is also evident, with altered expressions in several cancers (Esquela-Kerscher and Slack, 2006). Similar to that of regulators of gene expression, modulators of miRNA biogenesis are also coordinately regulated (Cai et al., 2009). In this context, the role of Wnt signaling in regulating miRNA biogenesis remains largely unknown, and of great interest.

As a strategy to identify potential miRNAs involved in the Wnt7a-dependent regulation of NSCLC cell growth, we performed miRNA expression profiling on Wnt7a-stimulated human lung adenocarcinoma cell line (A549) and identified hsa-miR29b as an important downstream target of Wnt7a. We show herein that hsa-miR29b expression is lost in NSCLC cell lines and Wnt7a-stimulation of NSCLC cell lines results in increased expression of hsa-miR29b. In addition, ERK5 and PPARγ, key effectors of Wnt7a/Fzd9 pathway, were also observed to be strong inducers of hsa-miR29b expression. Interestingly, knockdown of hsa-miR29b was enough to abrogate the tumor suppressive effects of Wnt7a and Fzd9 expression in NSCLC cells, suggesting that Wnt7a and/or hsa-miR29b plays a critical during lung tumorigenesis. Finally, we also show that hsa-miR29b plays an important role as a tumor suppressor in lung cancer by targeting murine double mutant 2 (MDM2), revealing novel nodes for Wnt7a/Fzd9-mediated regulation of NSCLC cell proliferation.

## Results

### Identification of Wnt7a regulated miRNAs in NSCLC cell lines

To identify potential miRNAs involved in Wnt7a-dependent regulation of NSCLC cell growth, we performed miRNA expression profiling of human lung adenocarcinoma cell line A549 as described in Materials and Methods (Table 1; supplementary material Table S1). A549s were of particular interest as they express Fzd9 but not Wnt7a, the ligand for Fzd9. The strategy was to transiently express either Wnt3 or Wnt7a in A549 cells, isolate total RNA, and screen for miRNA expression by using a cancer-specific miRNA super-array (Table 1; supplementary material Table S1). Interestingly, our screening succeeded in identifying hsa-miR29b as a novel miRNA regulated by Wnt7a. The reason for selecting hsa-miR29b over other miRNAs is 2-fold: 1) hsa-miR29b was upregulated by more than 19-fold in A549 cells expressing Wnt7a in comparison to empty vector control (Table 1), and 2) several studies have shown either a direct or an indirect role for hsa-miR29b in human cancers (Fabbri et al., 2007; Kole et al., 2011; Rothschild et al., 2012; Ru et al., 2012).

**Table 1.**
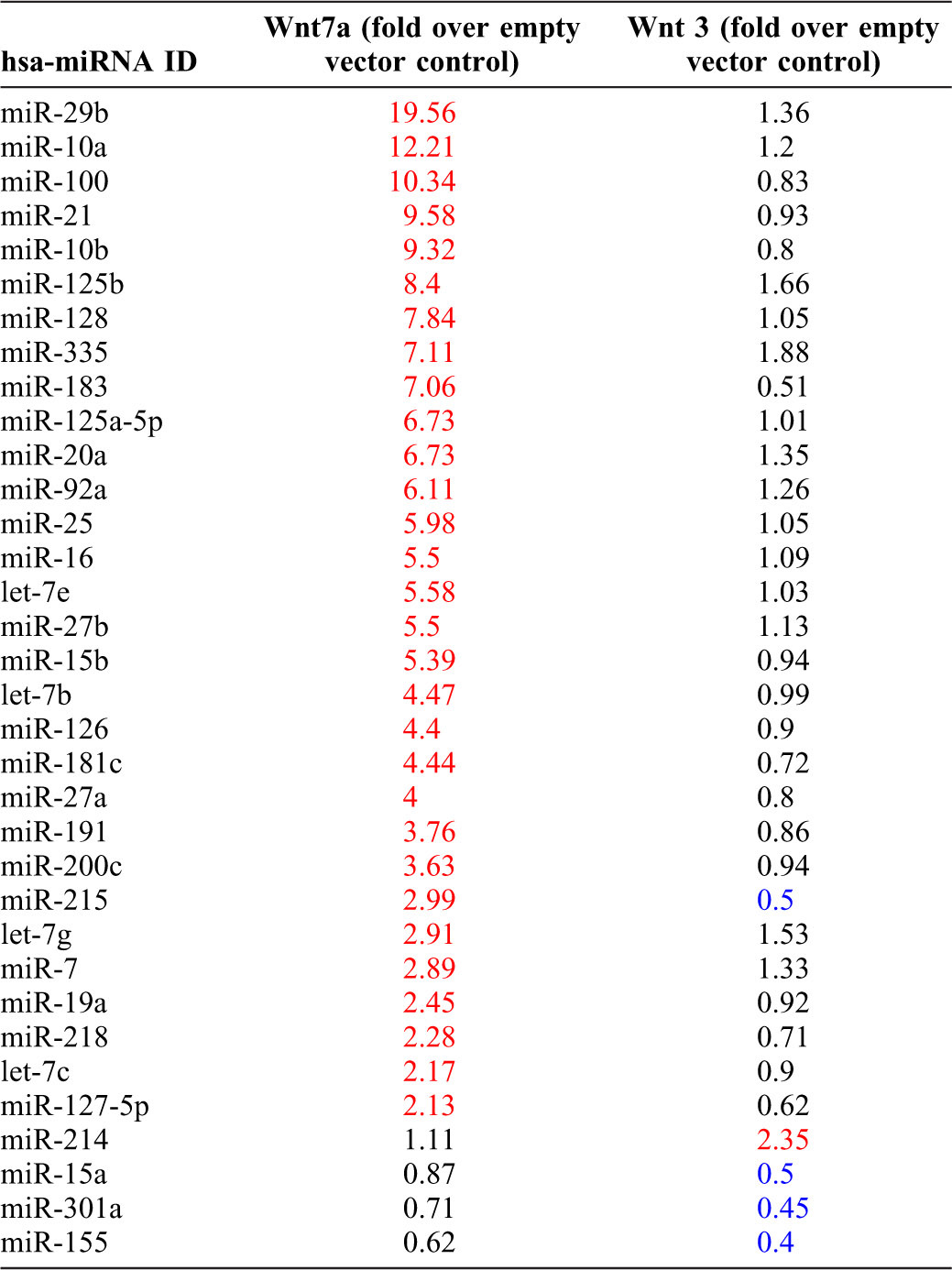
Wnt7a regulated miRNAs in A549 cells.

**Fig. 1.**
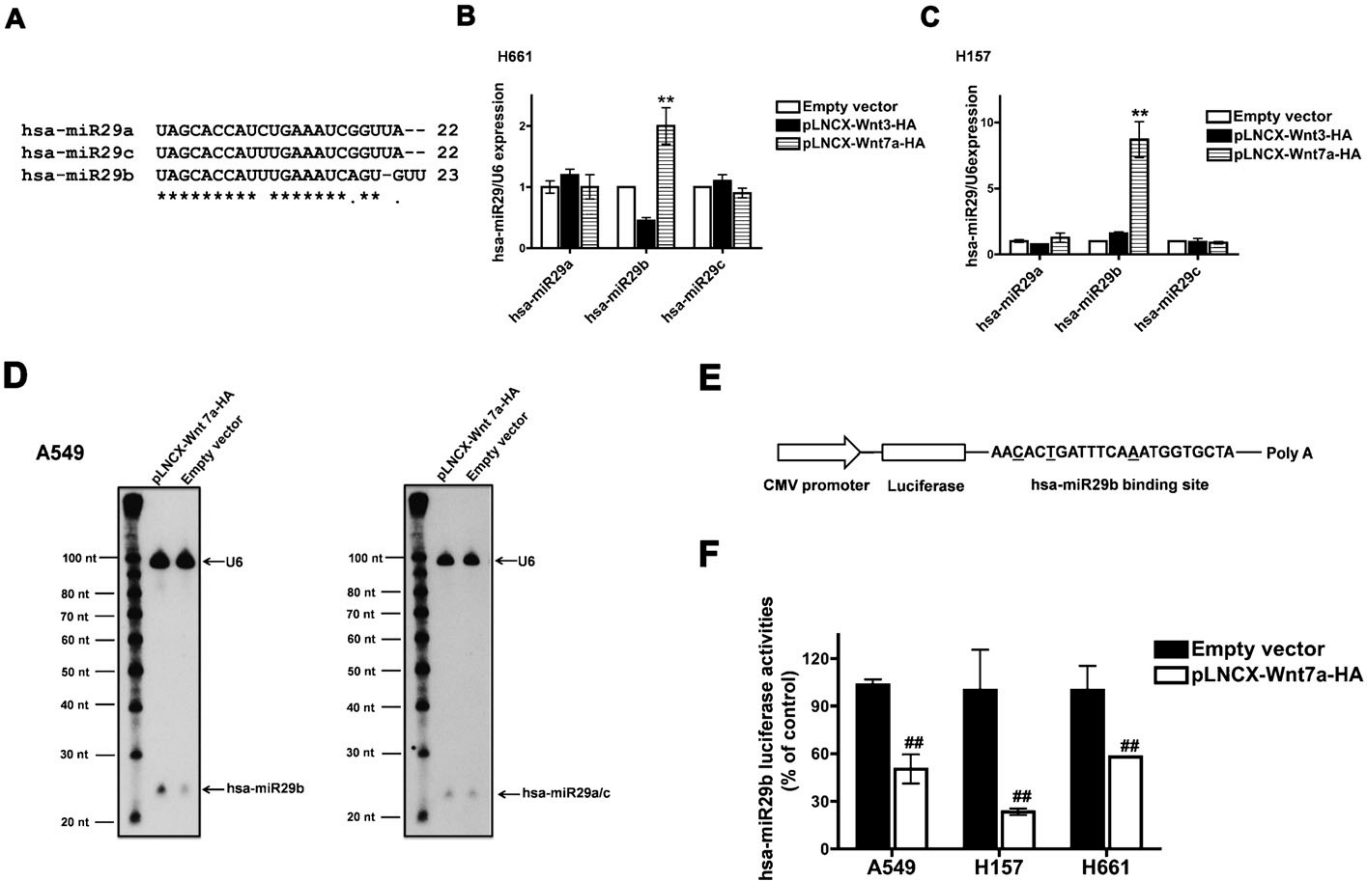
Wnt7a/Fzd9 signaling regulates hsa-miR29b. (**A**) Multiple alignments of hsa-miR29a, hsa-miR29b and hsa-miR29c. Real-time PCR analyses of the expression of hsa-miR29a, hsa-miR29b and hsa-miR29c in NSCLC cell lines. H661 (**B**) or H157 (**C**) cells were transfected either with empty vector, pLNCX-Wnt3-HA or pLNCX-Wnt7a-HA. After 24 h, total RNA was extracted and reverse transcribed. Real-time PCR analysis was carried out using the cDNAs and hsa-miR29a, hsa-miR29b or hsa-miR29c specific primers. RNU6B was used as the internal control for normalization. Data represent mean ± SEM of three separate experiments performed in duplicates. ^**^P<0.01; versus empty vector control. (**D**) Northern blot analysis of Wnt7a induced expression of hsa-miR29a, hsa-miR29b, hsa-miR29c and U6 in A549 cells. A549 cells were transfected with either empty vector or pLNCX-Wnt7a-HA. Total RNA was extracted and low molecular weight RNA was enriched, and Northern analysis was performed as described in Materials and Methods. (**E**) Schematic representation of hsa-miR29b luciferase reporter plasmid. The binding site of hsa-miR29b differs from that of hsa-miR29a or hsa-miR29c at the underscored bases. (**F**) hsa-miR29b luciferase reporter assay in NSCLC cell lines. A549, H157 and H661 cells were transfected either with empty vector or pLNCX-Wnt7a-HA along with hsa-miR29b luciferase reporter plasmid. After 48 h, the cells were lysed and luciferase activities were measured as described in Materials and Methods. H157 cells were also transfected with Fzd9, as they do not express Fzd9. Luciferase values were normalized to CMV-β-galactosidase values were represented in the graph. Wnt7a-induced hsa-miR29b expression is represented as the percentage of empty vector control. Data represent mean ± SEM of three separate experiments. ^##^*P*<0.01; versus empty vector control.

The hsa-miR-29 family includes three members: hsa-miR29a, hsa-miR29b and hsa-miR29c (Fig. 1A). It was interesting to note that Wnt7a induced the expression of only hsa-miR29b but not hsa-miR29a or hsa-miR29c (supplementary material Table S1). To corroborate our PCR array data, we performed q-RT-PCR analyses on RNAs isolated from two NSCLC cell lines (H661 and H157) transiently transfected with either empty vector, Wnt3, or Wnt7a expression vectors and by using primers specific for the hsa-miR29 family members (Fig. 1B,C). H157 cells were additionally transfected with Fzd9 as they have no expression of endogenous Fzd9 (H157+Fzd9). Consistent with our PCR array data, Wnt7a induced the expression of hsa-miR29b, but not hsa-miR29a or hsa-miR29c (Fig. 1B,C). Additionally, we also performed Northern blot analysis using ^32^P-labelled hsa-miR29b or hsa-miR29a/c specific probes to confirm the Wnt7a-induced hsa-miR29b expression (Fig. 1D). Consistent with our PCR array and q-RT-PCR data, Northern analysis revealed a robust Wnt7a-induced expression of hsa-miR29b, as detected by hsa-miR29b specific probes (Fig. 1D). On the other hand, probing the same blot with hsa-miR29a and hsa-miR29c specific probes (since there is only one base difference between the mature forms of hsa-miR29a and hsa-miR29c, we probed the blot with both the probes) showed no such increase in hsa-miR29a and hsa-miR29c expression (Fig. 1D). It was also interesting to note that Wnt7a stimulated the expression of only the mature form of hsa-miR29b but not its primary or precursor form (Fig. 1D). These data suggest that Wnt7a regulates hsa-miR29b, but not hsa-miR29a or hsa-miR29c.

We also interrogated Wnt7a-mediated regulation of hsa-miR29b by using an hsa-miR29b-specific luciferase reporter plasmid (Fig. 1E). In this reporter, the complimentary sequence of hsa-miR29b has been engineered downstream of luciferase gene (Fig. 1E). In cells expressing the reporter, mature hsa-miR29b targets the binding site downstream of luciferase gene resulting in repression in luciferase gene expression and as detected by reduced luciferase enzyme activity. Therefore, a decreased luciferase activity represents increased expression of hsa-miR29b and vice versa. Similar luciferase reporters have been widely utilized for miRNA studies (Bao et al., 2012; Xin et al., 2012; Yang et al., 2012). Consistent with our q-RT-PCR and Northern analysis, Wnt7a stimulation of three different NSCLC cells (A549, H157+Fzd9, and H661) expressing hsa-miR29b-luciferase reporter plasmid displayed a similar reduction in luciferase activities, strongly indicating an increased hsa-miR29b expression upon Wnt7a stimulation (Fig. 1F). Of note, Wnt7a-mediated induction of hsa-miR29b expression is unidirectional. Expression of hsa-miR29b, on the contrary, failed to impact Wnt7a expression (data not shown). In total, by using several distinct and powerful analyses we establish that activation of a β-catenin-independent pathway by Wnt7a stimulates the expression of hsa-miR29b, but not hsa-miR29a or hsa-miR29c, in NSCLC cells.

### hsa-miR29b regulates NSCLC cell proliferation

**Fig. 2.**
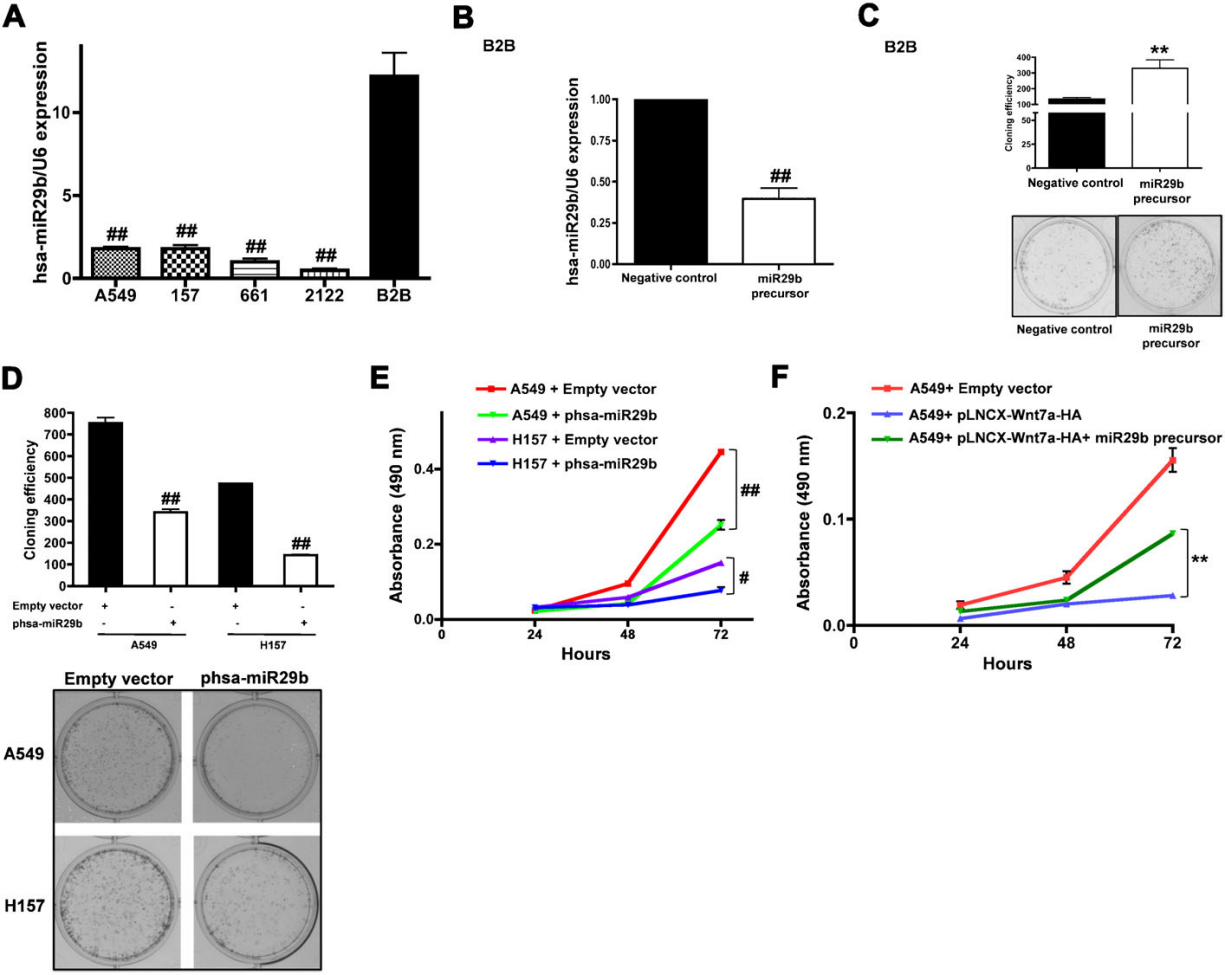
hsa-miR29b regulates NSCLC cell proliferation. (**A**) Real-time PCR analyses of the expression of hsa-miR29b in NSCLC cell lines and non-transformed cell lines. Total RNA was extracted from a non-transformed cell line (Beas2B) or NSCLC cell lines (A549, H157, H661 and H2122) and hsa-miR29b expression was quantified as described in Materials and Methods. RNU6B was used as the internal control for normalization. Data represent mean ± SEM of three separate experiments performed in duplicates. ^##^*P*<0.01; versus control (Beas2B). (**B**) Real-time PCR analyses of the extent of knockdown of hsa-miR29b in Beas2B cells. Beas2B cells were treated with either negative control or synthetic double stranded miR29b precursors. After 48 h, total RNA was extracted and the expression levels of hsa-miR29b were measured as described in Materials and Methods. RNU6B was used as the internal control for normalization. Data represent mean ± SEM of three separate experiments performed in duplicates. ^##^*P*<0.01; versus negative control. (**C**) Clonogenic assays were performed on Beas2B cells treated with miR29b precursors as described in Materials and Methods. Upper panel represents mean ± SEM of the number of colonies counted from two independent and highly reproducible experiments, while representative images were displayed in the lower panel. ^**^P<0.01; versus negative control. NSCLC cells (A549 or H157) were transfected with either empty vector or phsa-miR29b plasmid and cell proliferation rates were determined either by using a clonogenic assay (**D**) or an MTS assay (**E**) as described in Materials and Methods. Upper panel represents mean ± SEM from two independent highly reproducible experiments, while representative images were displayed in the lower panel. ^#^*P*<0.05; ^##^*P*<0.01; versus empty vector control. (**F**) A549 cells were transfected with or without pLNCX-Wnt7a-HA plasmid followed by treatment with miR29b precursors. Cell proliferation rates were later determined using an MTS assay as described in Materials and Methods. Data represent mean ± SEM from three independent highly reproducible experiments. ^**^P<0.01; versus A549+pLNCX-Wnt7a-HA.

Since, Wnt7a induce hsa-miR29b expression in NSCLC cell lines (Table 1; Fig. 1) and Wnt7a expression is lost in a majority of NSCLC cell lines (Winn et al., 2005), we probed next for the expression levels of hsa-miR29b in a panel of NSCLC cell lines using quantitative RT-PCR (qPCR, Fig. 2A). For these experiments, total RNA was extracted from normal lung bronchial epithelial cells (Beas2B), lung adenocarcinoma (A549, H2122), squamous cell carcinoma (H157+Fzd9) and large cell carcinoma cell lines (H661), reverse transcribed and the cDNAs were later used to measure the levels of hsa-miR29b expression (Fig. 2A). Q-PCR established the relative expression of hsa-miR29b in normal and NSCLC cell lines (Fig. 2A). Interestingly, hsa-miR29b expression was severely attenuated in all the NSCLC cell lines tested when compared to non-transformed bronchial epithelial cell line (Beas2B, Fig. 2A). Earlier, we have shown a loss in Wnt7a expression in a similar panel of NSCLC cell lines in comparison to short-term bronchial epithelial cell line [STBE (Winn et al., 2005)].

Since, hsa-miR29b expression was attenuated in all the NSCLC cell lines tested; we probed next if hsa-miR29b could affect NSCLC cell proliferation, either by using gene specific knockdowns (Fig. 2B,C) or re-expression of hsa-miR29b (Fig. 2D,E) in NSCLC cells. Since, normal bronchial epithelial cells (Beas2B) express high levels of hsa-miR29b (Fig. 2A), we first probed the effects of chemically synthesized double stranded miR29b precursor molecules (Ambion, anti-miR29b1 and anti-miR29b2) on Beas2B cell proliferation. Treatment of Beas2B cells with miR29b precursors showed a significant decrease in hsa-miR29b levels (>50%, Fig. 2B). Interestingly, depletion of hsa-miR29b in Beas2B cells revealed a significant increase in cell proliferation (by 3-fold), as detected by clonogenic assays (Fig. 2C). Consistent with the effects of hsa-miR29b knockdown on Beas2B cell proliferation, re-expression of hsa-miR29b was inhibitory to the cell growth of A549 or H157 cells as determined by clonogenic (Fig. 2D) or MTS cell proliferation assays (Fig. 2E).

Stimulation of NSCLC cells with Wnt7a not only induced the expression of hsa-miR29b but also attenuated NSCLC cell proliferation (Tennis et al., 2010; Winn et al., 2005). To test if the anti-proliferative effects of Wnt7a in NSCLC cells are mediated via the induction of hsa-miR29b, we first stimulated A549 cells with Wnt7a (to induce hsa-miR29b expression), followed by treatment with miR29b precursors. Interestingly, the expression of Wnt7a in A549 cells (that lack endogenous Wnt7a) severely attenuated A549 cell proliferation (Fig. 2F), whereas depletion of hsa-miR29b in A549 cells re-expressing Wnt7a blocked the inhibitory effects of Wnt7a expression on A549 cell growth (Fig. 2F). These data suggest that has-miR29b is a novel downstream target of Wnt7a/Fzd9 signaling and the anti-tumorigenic effects of Wnt7a in NSCLC cells.

### ERK5 and PPARγ modulate hsa-miR29b expression in NSCLC cells

**Fig. 3.**
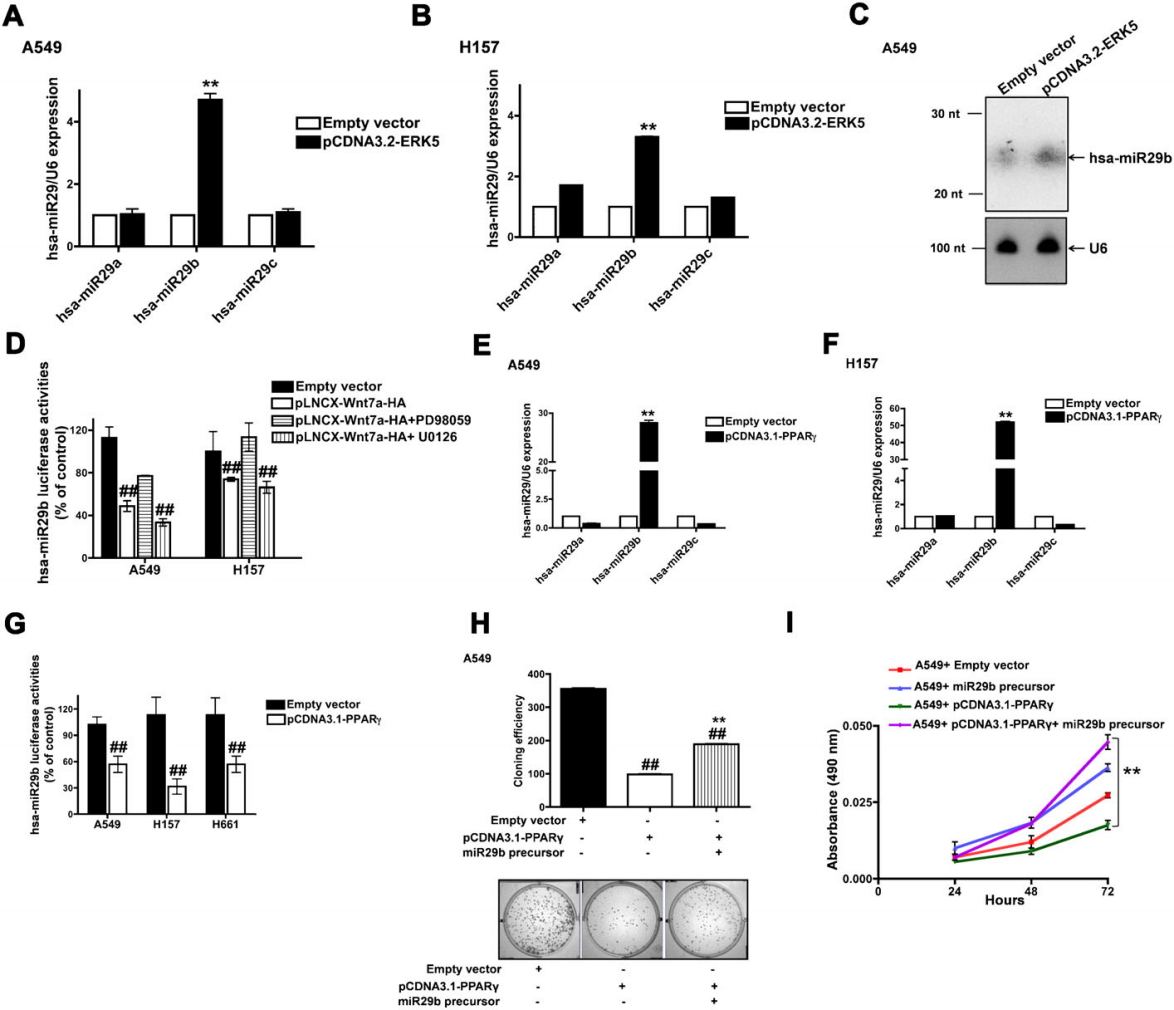
ERK5 and PPARγ stimulate hsa-miR29b expression in NSCLC cells. A549 (**A**) or H157 (**B**) cells were transfected either with empty vector or ERK5 expression plasmids. After 24 h, total RNA was extracted, reverse transcribed, and real-time PCR analysis was carried out using hsa-miR29a, hsa-miR29b or hsa-miR29c specific primers as described in Materials and Methods. RNU6B was used as the internal control for normalization. Data represent mean ± SEM of three separate experiments performed in duplicates. ^**^P<0.01; versus empty vector control. (**C**) Northern blot analysis of ERK5-induced expression of hsa-miR29b in A549 cells. A549 cells were transfected either with empty vector or ERK5 expression plasmids. After 24 h, total RNA extracted and low molecular weight RNA was enriched as described in Materials and Methods. 5 µg of low molecular weight RNA was separated on UREA-PAGE gel, and Northern analysis was performed as described in Materials and Methods. (**D**) A549 or H157 cells were transfected with Wnt7a expression vector together with hsa-miR29b luciferase reporter plasmid, followed by a treatment either without or with MEK inhibitors PD98059 (20 µM) or U0126, (10 µM). After 24 h, the lysates were assayed for luciferase activities as described in Materials and Methods. Data represent mean ± SEM of three separate experiments. ^##^*P*<0.01; versus empty vector control. A549 (**E**) or H157 (**F**) cells were transfected either with empty vector or PPARγ expression plasmids. After 24 h, total RNA was extracted, reverse transcribed, and real-time PCR analysis was carried out using hsa-miR29a, hsa-miR29b or hsa-miR29c specific primers as described in Materials and Methods. RNU6B was used as the internal control for normalization. Data represent mean ± SEM of three separate experiments performed in duplicates. ^**^P<0.01; versus empty vector control. (**G**) A549 or H157 cells were transfected either with empty vector or PPARγ plasmids along with hsa-miR29b luciferase reporter. After 24 h, the lysates were assayed for luciferase activities as described in Materials and Methods. Data represent mean ± SEM obtained from three independent experiments. ^##^*P*<0.01; versus empty vector control. (**H**) A549 cells were transfected with PPARγ expression plasmids either alone or together with miR29b precursors. After 24 h, the cells were seeded for clonogenic assays as described in Materials and Methods. Upper panel represents mean ± SEM of number of colonies counted from two independent experiments, while the lower panel displays representative images. ^**^P<0.01; versus A549+PPARγ, ^##^*P*<0.01; versus empty vector control. (**I**) A549 cells were transfected with PPARγ expression plasmids either alone or together with miR29b precursors. Cell proliferation rates were later determined using an MTS assay as described in Materials and Methods. Data represent mean ± SEM from three independent highly reproducible experiments. ^**^P<0.01; versus A549+PPARγ.

Wnt7a/Fzd9 signaling leads to activation of ERK5 and PPARγ and their associated effects on the inhibition of NSCLC growth (Winn et al., 2006). We therefore probed if ERK5 could also modulate hsa-miR29b expression (Fig. 3A,B). For these experiments, A549 or H157 cells were transfected with either empty vector or pCDNA3.2-ERK5 (Fig. 3A,B). Interestingly, Q-RT-PCR analyses of RNA isolated from ERK5 overexpressing NSCLC cells, revealed a 4-fold (A549) and a 3-fold (H157) increase in hsa-miR29b expression, but not hsa-miR29a or hsa-miR29c expression (Fig. 3A,B). We also confirmed that ERK5-induced hsa-miR29b levels in A549 cells via Northern analyses (Fig. 3C). In order to test the specificity of ERK5-mediated induction of hsa-miR29b, we made use of hsa-miR29b-specific luciferase reporter and MEK inhibitors, PD98059 and U0126 (Fig. 3D). For these studies, A549 or H157 cells expressing either hsa-miR29b luciferase reporter alone or together with Wnt7a-HA plasmid were treated without or with MEK inhibitors (Fig. 3D). H157 cells were additionally transfected with Fzd9 as they do not express endogenous Fzd9. Expression of Wnt7a, as expected induced hsa-miR29b expression as revealed by the reduced luciferase activities (Fig. 3D) in both the cell lines. Interestingly, treatment of Wnt7a expressing A549 or H157+Fzd9 cells with PD98059 [that blocks, MEK 1, 2 and 5, (Cameron et al., 2003; Kamakura et al., 1999; Winn et al., 2006)], blocked Wnt7a-induced hsa-miR29b expression, as revealed by an increase in luciferase activities (Fig. 3D). In strong contrast, treatment of Wnt7a expressing A549 or H157 cells with U0126 [that blocks only MEK 1 and 2 (Cameron et al., 2003; Kamakura et al., 1999; Winn et al., 2006)] has no impact on Wnt7a-induced hsa-miR29b expression (Fig. 3D), strongly suggesting that ERK5 (but not ERK 1 and 2) mediates Wnt7a-induced hsa-miR29b expression.

We next probed if the distal downstream effector of Wnt7a/Fzd9 pathway *viz*., PPARγ (Winn et al., 2006) could also regulate the expression of hsa-miR29b (Fig. 3E,F). Similar to the effects of ERK5 expression, overexpression of PPARγ in A549 or H157 cells also induced a 28-fold (A549, Fig. 3E) and 54-fold (H157, Fig. 3F) increase in hsa-miR29b expression, but not hsa-miR29a or hsa-miR29c expression, as determined by Q-RT-PCR (Fig. 3E,F). Consistent with our Q-RT-PCR analyses, PPARγ expression also induced hsa-miR29b expression in NSCLC cells (A549, H157 and H661), as revealed by reduced hsa-miR29b-luciferase activities (Fig. 3G). Furthermore, PPARγ-induced anti-proliferative effects on A549 cell growth was abrogated by the knockdown of hsa-miR29b, as determined by clonogenic assays (Fig. 3H) and MTS cell proliferation assay (Fig. 3I). These data strongly suggest that the anti-proliferative effects of PPARγ are also mediated via the induction of hsa-miR29b expression.

### hsa-miR29b regulates MDM2 expression

**Fig. 4.**
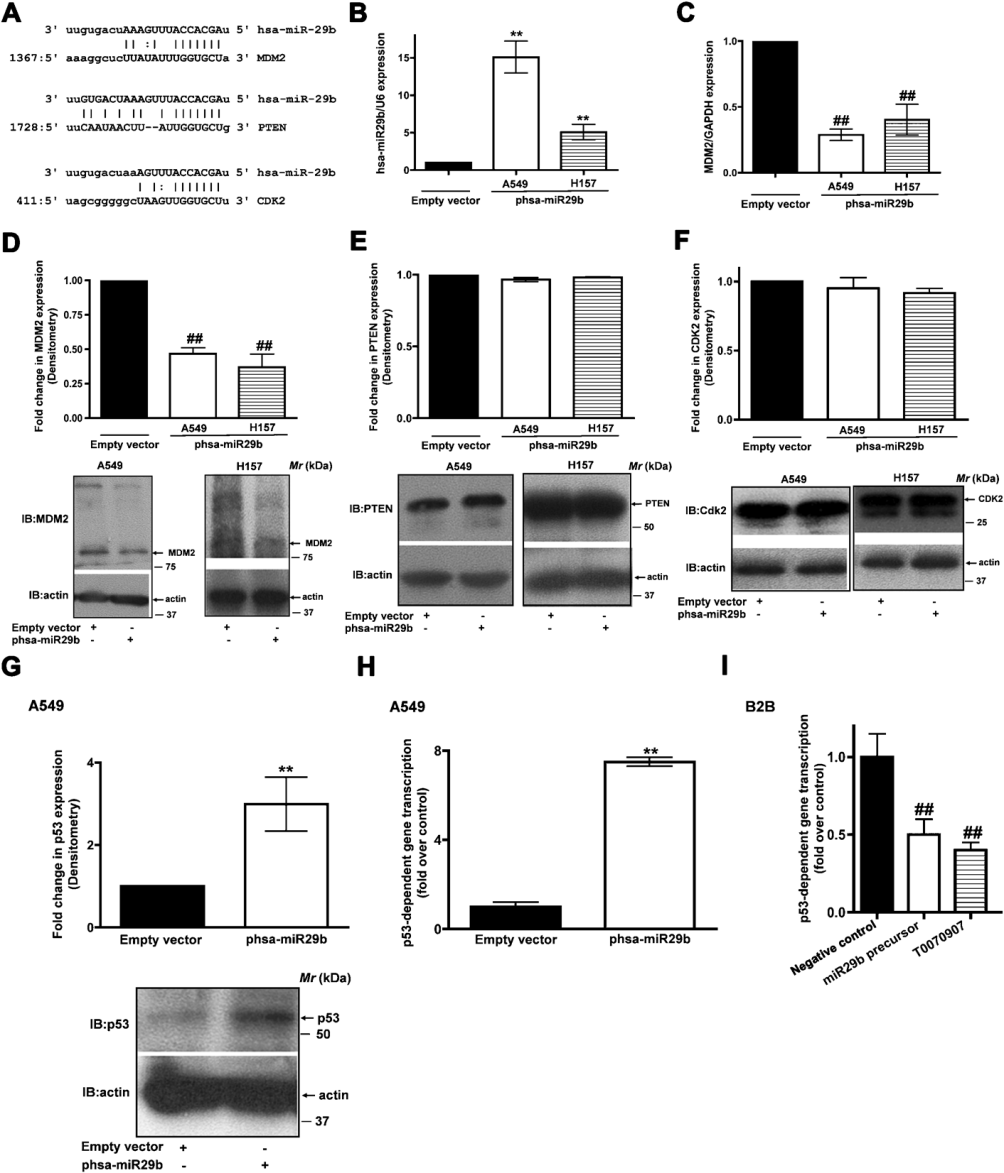
hsa-miR29b regulates MDM2 expression in NSCLC cells. (**A**) *In silico* identification of complimentary sites for hsa-miR29b on the 3′-UTR of MDM2, PTEN and CDK2. A549 or H157 cells were transfected either with empty vector or phsa-miR29b plasmid. After 24 h, total RNA was extracted, reverse transcribed, and real-time PCR analysis was carried out using hsa-miR29b specific primers (**B**) or MDM2 specific primers (forward: 5′-TTGACCTGTCTATAAGAGAATTATATATTTC-3′, reverse: 5′-GTCTTACGGGTAAATGGTGGCT-3′) (**C**). RNU6B and GAPDH were used as internal controls for normalization. Data represent mean ± SEM of three separate experiments performed in duplicates. ^**^P<0.01; versus empty vector control, ^##^*P*<0.01; versus empty vector control. A549 or H157 cells were transfected either with empty vector or phsa-miR29b plasmid for 24 h. The lysates were later immunoblotted for MDM2 (**D**), PTEN (**E**) and Cdk2 (**F**) expression. Upper panel represents mean ± SEM of the densitometry values of the immunoreactive bands from three independent highly reproducible experiments, while representative blots are displayed in the lower panels. ^##^*P*<0.01; versus empty vector control. (**G**) A549 cells were transfected either with empty vector or phsa-miR29b plasmid. After 24 h, the lysates were assayed for p53 expression by using anti-p53 antibodies. Upper panel represents mean ± SEM of the densitometry values of the p53 immunoreactive band from three independent highly reproducible experiments, while representative blots were displayed in the lower panels. ^**^P<0.01; versus empty vector control. (**H**) A549 cells were co-transfected either with empty vector or phsa-miRNA29b plasmid with p53-luciferase (Stratagene) reporter construct, with luciferase under the control of 14 repeats of p53-binding sequence (TGCCTGGACTTGCCTGG). After 24 h, the lysates were assayed for p53-dependent luciferase activities as described in Materials and Methods. Data represent mean ± SEM of normalized luciferase activities obtained from three independent experiments. ^**^P<0.01; versus empty vector control. (**I**) Beas2B cells were transfected either with negative control or miR29b precursors together with p53-luciferase reporter plasmid. After 24 h, the cells were treated either with or without PPARγ inhibitor (T0070907, 10 µM) as described in Materials and Methods. The cell lysates were later assayed for luciferase reporter expression as described in Materials and Methods. Data represent mean ± SEM of normalized luciferase activities obtained from three independent and highly reproducible experiments. ^#^*P*<0.05; ^##^*P*<0.01; versus empty vector control.

In order to identify potential hsa-miR29b targets, which are specific to lung cancer and tumor suppressor pathway, we scanned for hsa-miR29b targets *in silico* (http://www.microrna.org; Table 2). Among the several targets identified is the human homologue of murine double mutant 2, MDM2 (Fig. 4A). MDM2 is an important negative regulator of p53 tumor suppressor pathway (Oliver et al., 2011; Zhan et al., 2012). Since, hsa-miR29b expression in NSCLC cells is anti-proliferative, we hypothesize that expression of hsa-miR29b might downregulate MDM2 expression. We tested our hypothesis by measuring MDM2 transcript levels by Q-PCR in A549 and H157 cells upon re-expression of hsa-miR29b (Fig. 4B). In the presence of increased hsa-miR29b expression (Fig. 4B), we observed a corresponding decrease in MDM2 mRNA expression (by more than 50%) in both the cell lines tested (Fig. 4C). To further validate our findings, we also tested the effects of hsa-miR29b re-expression on MDM2 protein levels. Consistent to their effects on MDM2 mRNA, re-expression of hsa-miR29b in A549 or H157 cells (Fig. 4D) resulted in reduced MDM2 expression (Fig. 4D). To ascertain that the effects of hsa-miR29b expression on MDM2 were specific and that there were no off-target effects, we also tested the effects of hsa-miR29b re-expression on other proteins identified *in silico*, *viz*., phosphatase and tensin homologue deleted on chromosome Ten, PTEN (Fig. 4E) and Cdk2 (Fig. 4F). Although PTEN and Cdk2 display hsa-miR29b target sites (Fig. 4A), expression of hsa-miR29b in A549 or H157 cells had no impact either on PTEN (Fig. 4E) or Cdk2 (Fig. 4F) expression, suggesting that the observed effects of hsa-miR29b on MDM2 expression are indeed specific. Taken together, these data suggest that hsa-miR29b regulates NSCLC cell proliferation via repressing MDM2 expression.

**Table 2.**
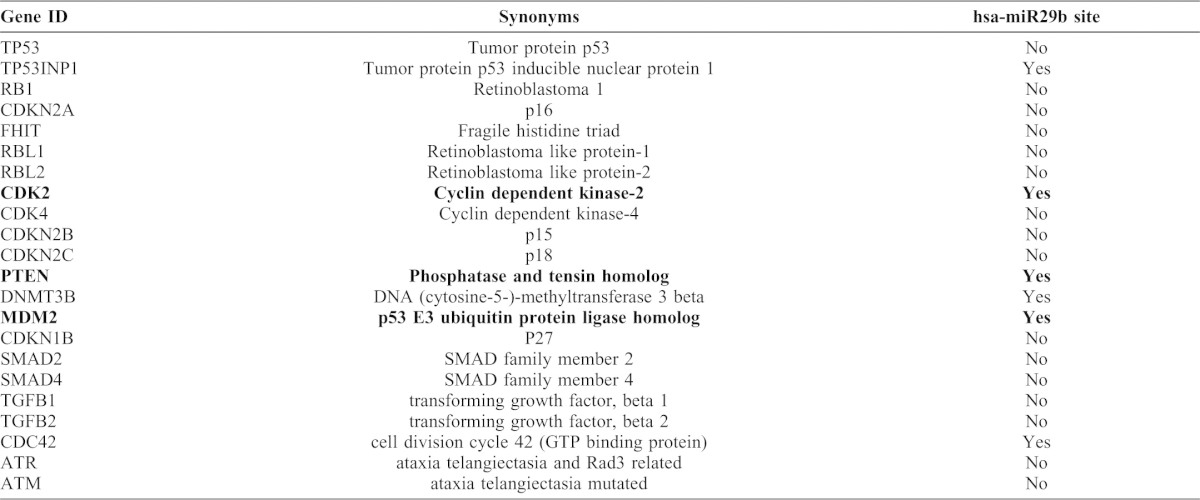
List of lung cancer-specific tumor-suppressor genes with potential hsa-miR29b targets.

Finally, since MDM2 is a known regulator of p53 tumor suppressor, we went further to probe the effects of hsa-miR29b expression on p53 expression (Fig. 4G,H). Consistent with the effects on MDM2 expression, expression of hsa-miR29b in A549 cells increased p53 expression as determined by western blots with anti-p53 antibodies (Fig. 4G). Since, p53 is a transcription factor that binds DNA and induce gene transcription, increased p53 expression (Fig. 4G) should result in increased p53-mediated gene transcription. To test this possibility, we made use of a p53-luciferase (Stratagene) construct, with luciferase under the control of 14 repeats of p53-binding sequence [TGCCTGGACTTGCCTGG (Wang et al., 2001)]. Co-expression of hsa-miR29b and p53-luciferase reporter in A549 cells resulted in an 8-fold induction in p53-dependent gene transcription in comparison to A549 cells transfected with p53-luciferase reporter alone (Fig. 4H). Consistent with the induction of p53-dependent gene transcription upon hsa-miR29b expression, knockdown of hsa-miR29b expression in non-transformed cells (Beas2B) resulted in reduced p53-dependent gene transcription (Fig. 4I), an effect similar to that of PPARγ inhibition (Fig. 4I) (Winn et al., 2006). In total, the results from several distinct and powerful analyses reveal a consistent story: Wnt7a stimulates hsa-miR29b expression and hsa-miR29b modulates NSCLC cell proliferation via repressing MDM2 expression.

## Discussion

Wnt7a is frequently lost in a subset of NSCLC (Winn et al., 2005), and restoration of Wnt7a expression in the context of Fzd9 results in increased differentiation and decreased transformed phenotype in cancer cell lines (Winn et al., 2005). In the current study, we identify hsa-miR29b as a novel tumor suppressor, which is regulated by Wnt7a in NSCLC cells. We show herein that the activation of a β-catenin-independent pathway, mediated by Wnt7a/Fzd9, strongly induce hsa-miR29b expression in NSCLC cells (Fig. 1), while activators of β-catenin-dependent pathway (Wnt3), in strong contrast, failed to stimulate hsa-miR29b expression. In addition, NSCLC cell lines displaying Wnt7a loss also showed an accompanying loss of hsa-miR29b expression (Fig. 2). These data strongly suggest that Wnt7a specifically regulates hsa-miR29b expression in the lung, and that loss of Wnt7a and/or hsa-miR29b might be an important player in the development of lung cancer.

Surprisingly, in the hsa-miR29 family, Wnt7a induced the expression of only hsa-miR29b, but not hsa-miR29a or hsa-miR29c. In strong agreement with our observations, recent studies also reveal specific induction of hsa-miR29b expression, but not hsa-miR29a or hsa-miR29c (Kole et al., 2011; Rothschild et al., 2012; Ru et al., 2012). In a more recent study, an important role for c-Src kinase in the regulation of hsa-miR29b was identified in human lung adenocarcinoma (Rothschild et al., 2012). The same study also identified an inhibitor of DNA binding/differentiation 1 (ID1) as a novel target for hsa-miR29b (Rothschild et al., 2012), in addition to DNA methyl transferase 3B [DNMT3B (Fabbri et al., 2007)]. Another study suggests increased efficacy of combination therapy of EGFR antibody with cisplatin/gemcitabine might be due to increased expression of hsa-miR29b and reduced expression of anti-apoptotic genes like DNA methyltransferease 3B (Samakoglu et al., 2012).

miRNAs offer precise gene regulation through post-transcriptional regulation of gene expression. However, the mechanism/s of regulation of miRNA expression is just beginning to emerge. We show herein for the first time that Wnt7a/Fzd9 signaling pathway in NSCLC cells leads to increased expression of hsa-miR29b (Fig. 1). We have shown previously that activation of Wnt7a/Fzd9 signaling pathway leads to activation of the mitogen activated protein kinase, ERK5 and subsequent activation of PPARγ (Winn et al., 2006). Interestingly, ERK5, obligate for Wnt7a-stimulated PPARγ activation, was also observed to be indispensable for hsa-miR29b expression. The ability of PPARγ to induce hsa-miR29b expression suggests that induction of hsa-miR29b expression is the most distal event of Wnt7a signaling. In prostate cancer, hsa-miR-143 was shown to interfere with ERK5 signaling (Clapé et al., 2009). Although miRNA-mediated regulation of ERK5 is evident, ERK5-mediated regulation of miRNAs has not been identified thus far. Our study provides the first evidence for an important role of a MAPK in regulating miRNA. In a similar manner, the role of PPARα in regulating miRNAs has been extensively studied (Shah et al., 2007), but the role/s of PPARγ on miRNA regulation remains largely unknown. Although a speculation, PPARγ might impose an indirect control on hsa-miR29b expression and regulate the biogenesis of mature form of hsa-miR29b, since Wnt7a or ERK5 could stimulate only the expression of mature form of hsa-miR29b (Figs 1, 3).

The current study also reveals a novel role for hsa-miR29b in MDM2 regulation. *In silico* analysis for hsa-miR29b complimentary sites identified MDM2 as a potential target (Fig. 4A). We confirmed our observation experimentally through hsa-miR29b expression, wherein expression of hsa-miR29b could block the expression of MDM2 both at the transcript level and protein level (Fig. 4). Similar effects for hsa-miR143/145 in regulating MDM2 have been reported (Zhang et al., 2013). These data suggest that loss of hsa-miR29b in cancers might lead to MDM2 upregulation and corresponding downregulation of p53 tumor suppressor. Indeed, re-expression of hsa-miR29b in NSCLC cells restored p53 expression and attenuated NSCLC cell proliferation (Fig. 4). A subset of NSCLC characteristically displays loss in Wnt7a (Winn et al., 2005), hsa-miR29b (current study) and p53 (Rom and Tchou-Wong, 2003), indicating that proper activation of Wnt7a signaling might be critical for p53 regulation and NSCLC cell proliferation.

**Fig. 5.**
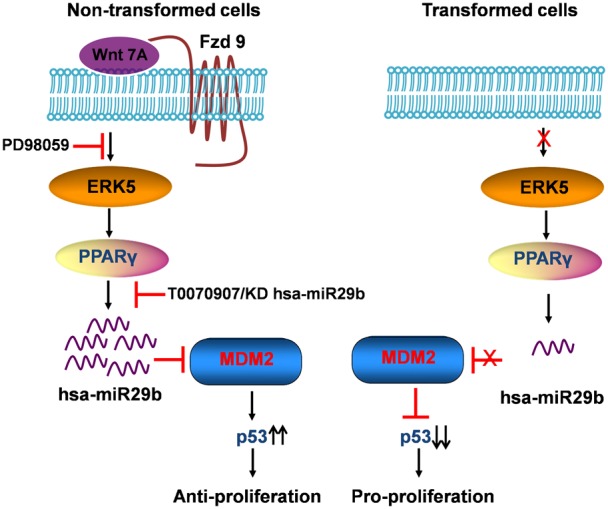
Schematic representation of the role of Wnt7a-induced hsa-miR29b expression in NSCLC proliferation. Wnt7a/Fzd9 signaling leads to induction hsa-miR29b, which is mediated by ERK5 and PPARγ. The hsa-miR29b expression targets MDM2 mRNA to degradation, which results in increased p53 levels and reduced cell proliferation. In NSCLC, on the contrary, absence of Wnt7a fails to activate Wnt7a/Fzd9 pathway, which blocks induction of hsa-miR29b expression. Loss in hsa-miR29b expression results in increased MDM2 levels reduced p53 expression and increased cell proliferation.

In summary, we propose herein a novel role for Wnt7a/Fzd9 signaling in inducing hsa-miR29b. Absence of Wnt7a in NSCLC fails to activate the Wnt7a/Fzd9 pathway, which in turn fails to induce hsa-miR29b expression. Furthermore, the loss of hsa-miR29b expression results in increased levels of MDM2, reduced p53 expression, and increased cell proliferation (Fig. 5). On the contrary, activation of Wnt7a/Fzd9 signaling by Wnt7a, and mediated by ERK5 and PPARγ, leads to the induction of hsa-miR29b. hsa-miR29b induction later promotes downregulation of MDM2, increased p53 expression, and reduced cell proliferation (Fig. 5). Thus, Wnt7a mediated regulation of hsa-miR29b represents a novel mechanism for Wnt7a/Fzd9-mediated regulation of NSCLC cell proliferation. Our data would also suggest that identifying pharmacological activators of Wnt7a/Fzd9 pathway and/or hsa-miR29b might have utility in the treatment of lung cancer.

## Materials and Methods

### Cell culture and inhibitors

NSCLC cell lines A549, H157 and H661 and a human non-transformed lung epithelial cell line (Beas2B) were cultured in RPMI 1640 medium (10-040-CV, Cellgro, Mediatech Inc., Manassas, VA) supplemented with 10% fetal bovine serum (FBS) in a humidified 5% CO_2_ incubator at 37°C. The cell lines were cultured bi-weekly and stocks of cell lines were passaged no more than ten times for use in experiments. The inhibitors used in our studies include, MEK inhibitors, [PD98059 (Sigma), U0126 (CalBiochem)] and PPARγ antagonist (T0070907, Calbiochem/EMD Biosciences). For miRNA expression studies, total RNA was isolated from NSCLC cells using TRIzol reagent (15596, Invitrogen, Carlsbad, CA) as per the manufacturer's instructions. To make cDNAs, miScript II RT kit (218161) and for qPCR, miScript SYBR Green PCR kit (218023) (Qiagen, Valencia, CA) were used. The qPCR primer for mouse miR29b (MPM00629A) was purchased from Qiagen (Qiagen, Valencia, CA).

### Clonogenic and MTS assays

Clonogenic assays were performed in triplicates by seeding 1×10^3^ cells in a well of 12-well culture plate followed by incubation at 37°C in a 5% CO_2_ incubator. After 72 h, cell colonies were stained using a staining solution (0.5% of Crystal Violet, 12% Gluteraldehyde, 87.5% of dd H_2_O) for 1 h at room temperature. After destaining in water and drying, colonies were quantified using Biorad Chemidoc Imaging System. Cloning efficiency represents the mean number of colonies formed per well.

MTS assays were performed in duplicates by seeding 500 cells in a well of 96-well culture plate, followed by incubation at 37°C in a 5% CO_2_ incubator. Cell proliferation was measured after 24, 48 and 72 h by adding 20 µl of MTS reagent (Cell Titer 96^®^ Aqueous One Solution, G3582, Promega Corporation, Madison, WI) to each well, followed by incubation at 37°C. After 1 h, the absorbance of the formazon product was measured at 490 nm using a plate reader. Normalized absorbance values (sample readings–readings of media only blank) were represented in the graphs.

### miRNA extraction and miRNA expression profiling

Total RNA was isolated from cell lines using TRIzol reagent (15596, Invitrogen, Carlsbad, CA) as per the manufacturer's instructions. cDNAs were made using miScript II RT kit (218161) and miScript SYBR Green PCR kit (218023, Qiagen, Valencia, CA) was used for qPCR. The qPCR primers for hsa-miR29a (MPH01244A), hsa-miR29b (MPH01245A) and hsa-miR29c (MPH01246A) were purchased from SA Biosciences (SA Biosciences Corporation, Frederick, MD) and RNU6 (243122) was purchased from Qiagen (Qiagen, Valencia, CA).

Expression levels of Wnt7a-stimulated miRNAs were measured by real-time RT-PCR using RT^2^ miRNA PCR Array System (MAH-102, SA Biosciences Corporation, Frederick, MD) as per the manufacturer's recommendations. Briefly, total RNA was extracted from A549 cells transiently transfected with either empty vector or Wnt 3 or Wnt7a expression plasmids. cDNAs were generated from the extracted total RNA and were later used as templates to carry out the miRNA expression profiling using pre-designed miRNA specific qPCR arrays. The miRNA PCR arrays used contained PCR primers for miRNAs, normalizer small RNAs and several quality controls. miRNA expression profiling and data analyses were carried out according to the manufacturer's instructions and is based on the ΔΔC_T_ method of relative quantification (supplementary material Table S1; Table 1). The miRNAs with a differential expression (fold change) of >2 are considered as upregulated and are highlighted in red, while miRNAs with fold change <0.5 are considered as downregulated and are highlighted in blue. The miRNAs that are upregulated only in the A549 cells treated with Wnt7a but not Wnt3 are considered as Wnt7a-specific miRNAs.

### Transfections and luciferase reporter assays

The reporter plasmids (hsa-miR29b-luciferase reporter and p53 luciferase-reporter), expression plasmids (pLNCX-Wnt7a-HA, pCDNA3.2-ERK5, pCDNA3.1-PPARγ and Fzd9) and CMV-β-galactosidase control plasmids were transiently transfected into NSCLC cells using LipofectAmine reagent (18324-012, Invitrogen, Carlsbad, CA, USA) as per the manufacturer's recommendations. The hsa-miR29b pcDNA plasmid was a kind gift from Dr Gregory Gores (Mayo Clinic). hsa-miR29b-luciferase reporter plasmid was purchased from Signosis (Cat. no. LR-0062), in which the mature hsa-miR29b complementary sequence was sub-cloned downstream of luciferase gene. Mature hsa-miR29b upon binding to its complimentary sequence in the reporter repress luciferase gene expression. Therefore, a decrease in luciferase activity represents increased expression of hsa-miR29b and vice versa. All of the luciferase activities were normalized to CMV-β gal activities. Wild-type pCDNA3.1-PPARγ plasmid was generously provided by Dr Rapheal Nemenoff (University of Colorado, Anschutz Medical Campus). p53-luciferase reporter, with luciferase under the control of 14 repeats of p53-binding sequence (TGCCTGGACTTGCCTGG) was obtained from Stratagene. The expression plasmid for pLNCX-Wnt7a-HA was a gift from Dr Jan Kitajewski (Columbia University) and pLNCX-Wnt3 was a gift from Dr Randall Moon (University of Washington). For studies involving the use of MEK inhibitors PD98059 (20 µM, Sigma) or U0126, (10 µM, Calbiochem/EMD Biosciences, San Diego, CA), A549 and H157 cells were co-transfected either without or with pLNCX-Wnt7a-HA and hsa-miR29b-luciferase reporter plasmids followed by treatment with MEK inhibitors. After 24 h, the lysates were assayed for luciferase activities. For PPARγ inhibitor studies, A549 cells were transfected with p53-luciferase reporter plasmid followed by treatment with PPARγ inhibitor T0070907 (10 µM, Calbiochem/EMD Biosciences, San Diego, CA). After 24 h, the lysates were assayed for luciferase activities. Beas2B cells were transfected with negative control precursors or chemically synthesized double stranded miR29b precursors (Ambion, 30 nM each of anti-hsa-miR29b-1 and anti-hsa-miR29b-2) together with p53 luciferase reporter plasmid. After 24 h, the cells were treated either with or without PPARγ inhibitor (T0070907, 10 µM) for another 24 h. The cell lysates were later analyzed for luciferase reporter expression.

### hsa-miR29b knockdown studies

Beas2B cells were seeded in a 12-well plate (6×10^4^ cells per well). After 16 h, cells were transfected with 30 nM each of chemically synthesized double stranded miR29b precursors [Ambion, anti-miR29b1 (AM1234) and anti-miR29b2 (AM12626)] or with a negative control (AM17010, Ambion Life Technologies, Grand Island, NY) using Attractene Transfection Reagent (301005, Qiagen, Valencia, CA). After 24 h, transfected cells were seeded into a 6-well plate (1×10^3^ cells per well) and incubated in a 37°C incubator. After 72 h, the cells were stained with clonogenic staining solution as described above. The sequences of the miRNA precursor inhibitors are as follows: Anti-miR29b1 (*GCUGGUUUCAUAUGGUGGUUUAGA*) and anti-miR29b2 (*CUGGUUUCACAUGGUGGCUUAG*).

### miRNA Northern blot analysis

Northern blots were performed as described earlier (Wang et al., 2011). Low-molecular-weight RNA were enriched with mirVana miRNA isolation kit (AM1560, Ambion Life Technologies, Grand Island, NY) with some modifications. 5 µg of small RNAs were separated on denaturing 12% polyacrylamide gels and transferred to Hybond N+ membrane (RPN303B, Amersham, GE Health care) using semi-dry electroblotting (Trans-Blot Semi-Dry transfer cell, BioRad). The membranes were ultraviolet cross-linked and baked at 80°C. The membranes were probed with 5′ end ^32^P-γ-ATP -labeled DNA oligonucleotides of either hsa-miR29b (*AACACTGATTTCAAATGGTGCTA*) or hsa-miR29a/c (*TAACCGATTTCAGATGGTGCTA* and *CCGATTTCAAATGGTGC*, since the mature sequences of hsa-miR29a/c differ in only one nucleotide, we used the probes together). Human U6 oligo (*GCAGGGGCCATGCTAATCTTCTCTGTATCG*) was used as an internal loading control probe.

### Immunoblot analysis

Proteins were extracted from the cell lysates using MAP kinase lysis buffer and the western blot analysis was carried out as previously described (Winn and Heasley, 2004). Anti-β-actin (3700), anti-p53 (2524) and anti-PTEN (9556) were purchased from cell signaling (Cell signaling Technology Inc., Danvers, MA). Anti-MDM2 (SC-812) and anti-Wnt7a (SC26361) were purchased from Santa Cruz Biotechnology (Santa Cruz Biotechnology, Santa Cruz, CA). Anti-Cdk2 (BD-610145) was purchased from BD Biosciences (BD Biosciences, San Jose, CA).

### Data analysis

Data were compiled from at least three independent, replicate experiments, each performed on separate cultures and on separate occasions. The responses are displayed as “fold-changes”. Comparisons of data among experimental groups were performed using student's *t*-test for assessing variance. Increase in statistical significance (*P* value of <0.05) is denoted with an “asterisk” symbol, while a decrease in statistical significance (*P* value of <0.05) is denoted with a “hash” symbol.

## Supplementary Material

Supplementary Material
