# Supplementary material for: hsa-miR29b, a critical downstream target of non-canonical Wnt signaling, plays an anti-proliferative role in non-small cell lung cancer cells via targeting MDM2 expression
Source: Biol Open. 2013 May 22;2(7):675–85. doi: 10.1242/bio.20134507 (PMC3711035; doi:10.1242/bio.20134507)
Supplement: Supplementary Material [file supp_2_7_675__index.html]

hsa-miR29b, a critical downstream target of non-canonical Wnt signaling, plays an anti-proliferative role in non-small cell lung cancer cells via targeting MDM2 expression — Supplementary Material 

# hsa-miR29b, a critical downstream target of non-canonical Wnt signaling, plays an anti-proliferative role in non-small cell lung cancer cells via targeting MDM2 expression

## bio.20134507 Supplementary Material

**Files in this Data Supplement:**

- Supplementary Material - Sreedevi Avasarala et al. doi: 10.1242/bio.20134507
