# Supplementary material for: hsa-miR29b, a critical downstream target of non-canonical Wnt signaling, plays an anti-proliferative role in non-small cell lung cancer cells via targeting MDM2 expression
Source: Biol Open. 2013 May 22;2(7):675–85. doi: 10.1242/bio.20134507 (PMC3711035; doi:10.1242/bio.20134507)
Supplement: Supplementary Material [file supp_bio.20134507_bio.20134507-s1.pdf]

Supplementary Material

Sreedevi Avasarala et al. doi: 10.1242/bio.20134507

Table S1. PCR array analysis for miRNAs regulated by Wnt7a in A549 cells.

hsa-miRNA ID	Fold upregulation		
	Control	Wnt 3	Wnt 7A
hsa-miR-335	5.03	1.88	7.11
hsa-miR-135b	2.79	1.34	1.51
hsa-let-7a	0.13	675.59	35.51
hsa-miR-125a-5p	27.28	1.01	6.73
hsa-miR-92a	0.1	1.26	6.11
hsa-miR-184	0.05	1.09	1.42
hsa-miR-214	0.05	2.35	1.11
hsa-miR-20a	14.03	1.35	6.73
hsa-let-7g	0.57	1.53	2.91
hsa-miR-29b	0.31	1.36	19.56
has-RNU6-2	1.23	1.13	1.6
hsa-miR-133b	0.05	−1.11	1.42
hsa-let-7b	0.07	−1.01	4.47
hsa-miR-205	0.08	−1.69	1.31
hsa-miR-181a	0.07	−1.38	1.15
hsa-miR-199a-3p	0.05	−1.09	1.59
hsa-miR-140-5p	1.6	−1.11	1.42
hsa-miR-193b	0.34	−1.46	1.21
hsa-miR-183	0.26	−1.96	7.06
hsa-miR-30c	7.73	−1.35	1.79
hsa-miR-148a	0.37	−1.11	1.42
hsa-miR-134	0.11	−1.47	1.47
hsa-miR-138	1.18	−1.16	1.73
hsa-miR-373	0.05	−1.16	1.01
hsa-let-7c	0.08	−1.11	2.17
hsa-let-7e	0.91	1.03	5.58
hsa-miR-218	1.29	−1.41	2.28
hsa-miR-21	1176.27	−1.08	9.58
hsa-miR-181d	0.11	−1.45	1.21
hsa-miR-200c	0.48	−1.06	3.63
hsa-miR-100	200.85	−1.21	10.34
hsa-miR-10b	41.93	−1.25	9.32
hsa-miR-1	0.36	−1.11	1.42
hsa-miR-363	0.05	−1.11	1.42
hsa-let-7i	0.32	−1.44	1.47
hsa-miR-27b	106.89	1.13	5.5
hsa-miR-7	14.52	1.33	2.89
hsa-miR-127-5p	0.11	−1.6	2.13
hsa-miR-191	2.89	−1.16	3.76
hsa-let-7d	0.06	−1.27	1.28
hsa-miR-9	5.43	−1.39	1.74
hsa-let-7f	0.15	−1.11	1.42
hsa-miR-10a	172.45	1.2	12.21
hsa-miR-181b	0.07	−1.45	1.01
hsa-miR-15b	6.41	−1.06	5.39
hsa-miR-16	83.29	1.09	5.5
hsa-miR-210	0.11	1.03	1.12
hsa-miR-17	5.28	−1.58	1.15
hsa-miR-25	19.56	1.05	5.98
hsa-miR-144	0.13	−1.11	1.42
hsa-miR-128	0.37	1.05	7.84
hsa-miR-215	2.83	−2.01	2.99
hsa-miR-19a	21.71	−1.09	2.45
hsa-miR-193a-5p	0.13	−1.27	1.17
hsa-miR-18a	1.39	−1.62	1.44
hsa-miR-125b	48.5	1.66	8.4
hsa-miR-126	67.18	−1.11	4.44
hsa-miR-27a	31.12	−1.25	4
hsa-miR-149	0.07	−1.11	1.42
hsa-miR-23b	48.5	−1.11	1.42
hsa-miR-203	0.08	−1.11	1.42
hsa-miR-32	1.38	−1.11	1.42
hsa-miR-181c	0.08	−1.39	4.44

Table S1. Continued.

hsa-miRNA ID	Fold upregulation		
	Control	Wnt 3	Wnt 7A
SNORD47	0.4	−1.11	1.42
hsa-miR-196a	0.45	−1.17	1.56
hsa-miR-142-5p	0.11	−1.11	1.42
hsa-miR-96	1.17	−1.11	1.42
hsa-miR-148b	0.35	−1.25	1.27
hsa-miR-18b	0.08	−1.39	1.39
**miRNA ID**	**Fold downregulation**		
	**Control**	**Wnt 3**	**Wnt 7A**
hsa-miR-122	0.03	−1.65	−1.04
hsa-miR-20b	1.88	−1.06	1.67
hsa-miR-222	0.06	−1.21	−1.13
hsa-miR-15a	1	−2.01	−1.15
hsa-miR-378	0.44	−1.57	−1.47
hsa-miR-146b-5p	0.17	−1.8	−1.14
hsa-miR-34c-5p	0.15	−1.65	−1.27
hsa-miR-146a	0.52	−1.51	−1.16
hsa-miR-212	0.08	−1.55	−1.09
hsa-miR-206	0.07	−1.18	−1.11
hsa-miR-124	0.07	−1.54	−1.06
hsa-miR-301a	2.06	−2.22	−1.4
hsa-miR-155	0.06	−2.53	−1.6
hsa-miR-150	0.07	−1.47	−1.21
hsa-miR-29a	2.46	−1.43	−1.3
hsa-miR-98	0.18	−1.91	−1.03
hsa-miR-34a	0.05	−1.73	−1.71
hsa-miR-143	0.06	−1.65	−1.19
hsa-miR-372	0.06	−1.55	−1.04
SNORD48	0.05	−1.49	−1.19
SNORD44	43.11	1.46	−1.92
